# Evaluation of Serum Testosterone, Progesterone, Seminal Antisperm Antibody, and Fructose Levels among Jordanian Males with a History of Infertility

**DOI:** 10.1155/2010/409640

**Published:** 2010-12-01

**Authors:** Hala I. Al-Daghistani, Abdul-Wahab R. Hamad, Muna Abdel-Dayem, Mohammad Al-Swaifi, Mohammad Abu Zaid

**Affiliations:** ^1^Department of Medical Allied Sciences, Al-Salt University College, Al-Balqa Applied University, Al-Salt 19117, Jordan; ^2^Department of Medical Allied Sciences, Zarka University College, Al-Balqa Applied University, Zarka 13115, Jordan; ^3^Medical Hussein City Hospital, Amman 11855, Jordan

## Abstract

Due to the biochemical complexity of seminal fluid, we attempt to study the possible correlation between fructose, which is secreted under the effect of androgen hormone, and autoimmunity, which might play a role in varicocele associated infertility, in reducing sperm motility. Seminal fructose, antisperm antibodies (ASAs) and blood steroids hormones (testosterone and progesterone) levels were measured in 66 infertile males with varicocele and 84 without varicocele referred for fertility treatment. Seminal analysis was performed with biochemical measurements of seminal fructose and mixed agglutination reaction (MAR) for ASA. Serum levels of progesterone and testosterone were estimated using a competitive chemoluminescent enzyme immunoassay. The mean values for serum testosterone were 380.74 ± 24.331, 365.9 ± 16.55, and 367.5 ± 21.8 ng/dl, progesterone 0.325 ± 0.243, 0.341 ± 0.022, and 0.357 ± 0.0306 ng/ml, and seminal plasma fructose 359.6 ± 26.75, 315.6 ± 13.08, and 332.08 ± 24.38 mg/dl in males with varicocele, without varicocele, and fertile males, respectively. A significant high level of testosterone was observed within varicocele group (*P* = .001). This result showed that testosterone may play a role as an infertility determinant in subjects with varicocele. ASA was detected in 18 (26.47%) of cases with varicocele, 20 (38.46%) without varicocele, and in 16 (32.0%) fertile men. Cases with ASAs associated with low sperm motility morphology. An inverse correlation between sperm-bound antibodies and viscosity has been shown (*P* = .017). ASA showed some significant inverse relations with ages, durations of infertility, and viscosity (*P* < .05). In addition, a significant correlation was observed between ASA positive seminal plasma and testosterone concentration among infertile cases (with or without varicocele) and fertile (*P* < .05). Our results suggest a relationship between testicular steroid hormone levels with autoimmunity and sperm antibodies which influence the motility of ejaculated spermatozoa among Jordanian infertile males.

## 1. Introduction

Infertility is defined as inability of couples to achieve pregnancy following one year of unprotected intercourse. By this criterion, infertility affects 13%–18% of couples and male factors account for up to half of all cases [1]. One of male infertility causes is varicocele which is present in 2%–22% of the adult male population [2]. In men with abnormal semen analysis, the prevalence of varicocele reached 25% [3]. Cases of varicocele have been linked to a serious of events such as: biochemical changes in the epididymal fluid, a stasis of the internal spermatic vein, elevated scrotal temperature, testicular hypoxia, and retrograde blood flow of renal and adrenal metabolites [4]. Immunological and hormonal factors are vital factors responsible for reduction in sperm motility. They appeared to have certain role in varicocele-related infertility [5]. Data about their influence on seminal fluid parameters are contradictory, since males with varicocele showed infertility with variable semen finding. Moreover, some varicose males appeared fertile, but their fertility potential might decline gradually [6]. Hormonal imbalance and sperm autoimmunity have been considered as two systems that work in close association and affecting each other [7–9]. However, few studies revealed no correlation between autoimmunity and hormonal factor [10].

Autoantibodies to sperms are present approximately in 10% of infertile males [11] and in (24.6% and 32%) among patients with varicocele [12, 13]. However, a number of investigators have found no association between ASA formation and varicocele [14, 15]. ASA impair the fertilizing ability of spermatozoa by acting negatively on sperm motility and result in poor cervical mucus penetration and in vitro gamete interaction [16]. Sperm-bound antibodies have been found to impair sperm function only when the degree of antibody binding is very high (>50%) [17]. 

Biochemical evaluation of seminal fluids suffering from varicocele provided some evidence on reduced fertility of their gametes. Any change in the biochemical composition of semen, such as reduced fructose levels, was known to cause a reduction in sperm motility [18]. Fructose is an important source of energy for the sperm. It is the principle source of the sperm motility under anaerobic conditions [19]. Lowered intensity of fructose oxidation in gamete mitochondria leads to accumulate lactate and inhibition of dehydrogenases activity [20]. This sugar has been studied extensively, because it is considered as a marker for seminal vesicle function [21]. Attempts to correlate fructose concentrations in seminal plasma with andrological parameters have produced inconsistent results. The content of fructose in fresh semen depends upon the secretion function of accessory glands which is influenced directly by the activity of the male sex hormone. The impaired sex accessory gland functions could arise from decreased venous drainage in the vesicoprostatic plexus. A low level of seminal fructose may coincide with other symptoms of hormonal malfunction and poor quality of spermatozoa. In varicocele, the impaired sex accessory gland secretions could themselves influence the motility of ejaculated spermatozoa [22]. However, other studies showed that seminal fructose did not have any statistically significant differences when comparing infertile patients with varicocele and fertile [23, 24]. 

Steroid hormones, such as testosterone, are necessary for the development and maintenance of secondary sexual characteristics as well as initiation and maintenance of spermatogenesis. It was known that males with varicocele and abnormal seminal fluids have lower concentrations of the testosterone hormone [25]. In addition, the secretion products of the secondary sex glands were more often in the lower range in the ejaculates of men combining varicocele with sexual disturbance, proving the decreased testosterone level to induce a deficient function of these glands [26]. Some relationships were also found between levels of testosterone and progesterone in the seminal plasma of semen samples with low sperm motility [7].

The impact of fructose, sperm autoantibodies, along with hormonal imbalance in the pathogenesis of varicocele is not known. In the present study, steroid hormones (testosterone and progesterone), ASA, and fructose were measured in serum and seminal plasma of infertile males with and without varicocele to study the possible correlation between the three parameters in reducing sperm motility.

## 2. Materials and Methods

### 2.1. Study Groups

One hundred and fifty infertile males (sixty eight (34%) with varicocele and eighty two (41%) without varicocele) were enrolled in the study with mean age of 31.87 ± 0.468 years and duration of infertility of 3.37 ± 0.236 years. Men were attending the infertility department at Medical Hussein City Hospital in Jordan, during December 2009 to January 2010 with complete medical and clinical histories. Patients were married and infertile (with their fertile female partner) for at least more than one year of unproductive intercourse. Varicocele was diagnosed after physical examination, duplex, and Color Doppler Ultrasonography. All cases of varicocele were classified as grade I (subclinical, audible, not visible, and not palpable) [27]. Control group consists of 50 (25%) fertile married males with mean age 33.0 ± 0.884 years. They are clinically asymptomatic males without varicocele and normal seminal fluids. Informed consent was obtained from all study cases.

### 2.2. Seminal Fluid Samples

Semen samples were obtained by masturbation after 3–5 days of sexual abstinence and kept in sterile nontoxic recipients. Written and verbal advices were given to the patients to follow the procedure. Each patient provided at least two samples within one month. Samples were allowed to liquefy in the incubator directly and analyzed for sperm volume, concentration, pH, viscosity, morphology, motility, and viability as indicated by the WHO manual for semen analysis [28]. Morphology was determined after incubation of the sample with trypsin for 10 minutes at 25°C according to the methylene blue eosin staining procedure, feathering, and fixation by flame. At least 100 cells were examined at a final magnification of 1000x. Viscosity of the liquefied sample was estimated by introducing a glass rod into the sample and observing the thread that forms on withdrawal of the rod. Threads obtained from normal samples should not exceed 2 cm in length [29]. Motility was expressed as a percentage of motile spermatozoa and their mean velocity. For the purpose of conventional analysis, a simple classification system is recommended in which a fixed volume of semen is delivered onto a clean glass slide and covered with a 22 × 22 mm cover slip [28]. The preparation is then examined at a magnification of 400x. The microscopic field is scanned systematically, and the motility of each spermatozoon encountered is graded a, b, c, or d. At least 100 spermatozoa are classified in this way. The presence of 50% or more with forward progression (categories a and b) or 25% or more with rapid progression (category a) within 60 minutes of ejaculation were considered as normal results. The results were averaged for the two samples, and a single value was used for each parameter. Sperm motility was calculated by multiplying sperm concentration (x10(6)/ml) and semen volume (ml).

### 2.3. Mixed Antiglobulin Reaction (MAR) Test

The MAR test is performed by mixing fresh, untreated semen with sheep blood cells (SRBs) coated with human IgG. A monospecific antihuman-IgG antiserum is added to this mixture, which was mixed and read within 10 minutes. Positive and negative control samples were run along with each experiment. The formation of mixed agglutinates between sRBC and motile spermatozoa proves the presence of IgG antibodies on the sperms [30]. Immunologic infertility is suspected when 10%–90% of the motile spermatozoa attached to the RBCs.

### 2.4. Determination of Seminal Fluid Fructose

The method is adopted from that of Seliwanoff. The principle depends upon the presence of fructose (ketoses), which forms a pink color when heated with resorcinol in the presence of hydrochloric acid (ARCOMEX, Fructose. S.F). The intensity of the red complex is proportional to the fructose concentration and measured photometrically at 490 nm [31].

### 2.5. Hormone Estimation

Serum levels of progesterone and testosterone were estimated by a competitive chemoluminescent enzyme immunoassay using IMMULITE 2000 Progesterone and IMMULITE 2000 Total Testosterone which utilized specific antibody-coated polystyrene beads as a solid phase. [32, 33]. After the sample was incubated with alkaline phosphatase-labeled regent, the bound label was then quantified using a specific chemoluminescent substrate and light emission will be detected by photomultiplier tube, and the results were calculated for each samples. The normal ranges for progesterone is 0.27–0.9 ng/ml and for testosterone 262–1593 ng/dl.

### 2.6. Statistical Analysis

SPSS software version 13.0 was used for data analysis. Results were presented as means value with deviations (±SD). Significance of the differences was performed using *t*-test for equality of means, ANOVA correlation, descriptive, frequency, and chi-squared test. A *P*-value of <.05 was considered.

## 3. Results

### 3.1. Seminal Fluid Parameters

Serum and seminal plasma obtained from 68 (34%) infertile males with varicocele and 82 (41%) without varicocele, and 50 (25%) fertile control males were investigated for the possible relations between fructose levels, steroid hormones, and autoimmunity to sperm antigens. Among the patients studied, the age varied from 25 to 47 years, thus covering the entire span of the reproductive years.

Analyzing of seminal fluid samples revealed significant differences in sperm viability, motility, and morphology (*P* ≤ .05) among our study groups (Table 1).

### 3.2. Fructose and Sperm Antibodies

 The level of fructose in the seminal plasma was (means ± SD)**: **359.6 ± 26.75 (range, 102–1038), 315.6 ± 13.08 (range, 35–671), and 332.08 ± 24.38 (range, 113–909) mg/dl in males with varicocele, without varicocele, and fertile males, respectively. The level of seminal fructose among infertile groups (with or without varicocele) was found to be slightly higher than fertile, without being statistically significant.

### 3.3. Antisperm Autoantibodies (ASAs)

Mixed Antiglobulin reaction test was used for sperm antibodies in the seminal plasma. ASA was detected in 18 (26.47%) male with varicocele, 20 (38.46%) without varicocele, and in 16 (32%) fertile males. Most of tested samples showed ≥40% of the motile sperm which have been attached to RBCs. No significant difference in ASA was recorded between males with varicocele and other without in comparison to fertile males. ASA showed some significant inverse relation with ages, durations of infertility, and viscosity (*P* < .05). Most semen samples positive for ASA 50 (92.6%) showed normal viscosity, while only 4 (7.4%) semen with autoantibodies appeared viscous (Table 2).

### 3.4. Steroid Hormone

**Figure 1:**
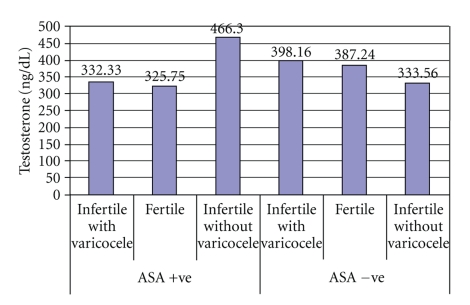
Testosterone levels in the serum of infertile and fertile men in relation to sperm antibodies.

**Figure 2:**
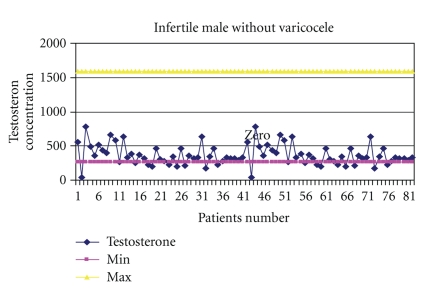
Ranges of testosterone concentration in the serum of infertile males without varicocele.

**Figure 3:**
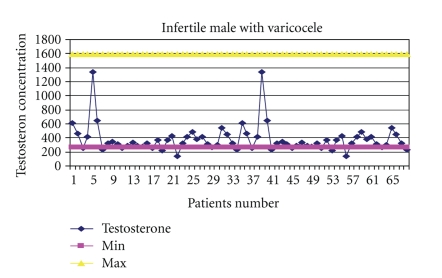
Range of testosterone concentrations in the serum of infertile males with varicocele.

**Figure 4:**
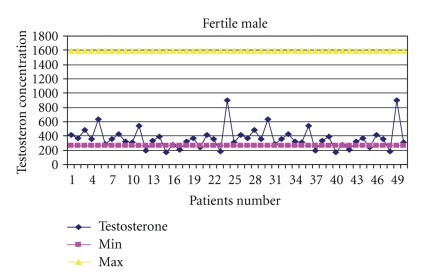
Ranges of testosterone concentration in the serum of fertile males.

The mean values for serum testosterone and progesterone were: (means ± SD) 380.74 ± 200.6 (range, 140–1342), 365.9 ± 149.9 (range, 173–903), and 367.5 ± 154.2 (range, 42–788) ng/dl for testosterone, while 0.325 ± 0.200 (range, 0.15–0.86), 0.357 ± 0.216 (range, 0.15–1.10), and 0.341 ± 0.208 (range, 0.09–0.89) ng/ml for progesterone in males with varicocele, without varicocele, and fertile males, respectively (Table 1). The levels of progesterone was found to increase with ages (*P* = .007). Correlation results revealed a significant difference in testosterone concentration between infertile and fertile group (*P* = .047), with the highest level in males with varicocele (Figures 2, 3, and 4). Progesterone level was found to have a direct significant relation to the serum level of testosterone (*P* = .008). Analysis of variants showed a significant correlation between antibodies to sperm present in the seminal fluids of infertile and fertile men and steroid levels such as testosterone in their serum (*P* < .05) (Figure 1).

## 4. Discussion

It has been found that prostate and vesicle infection and subclinical reproductive tract infection may lead to dysfunction of sperm and changes in semen parameters, and the latter may consequently lead to infertility. Some possible pathophysiological mechanisms of the development of infertility are linked either to inhibition of spermatogenesis resulting from testicular damage or autoimmune process [34, 35]. Varicocele is the most common cause of male infertility and its pathophysiology is multifactorial. 

Two hundred Jordanian males form in this study under investigation. The number of patients aged between 25 to 45 years was very high. This might be due to the fact that in our society, the majority of marriages take place in this age group. The least number of cases were seen in age groups 47 and beyond, as late marriages are not common in our society. 

The function of sex accessory glands in infertile males and fertile males was investigated by determining fructose levels in the seminal fluids. Regarding fructose estimation, it was noted from this study that fructose concentration did not differ significantly between the whole infertile group and the fertile group, beside that, there was no significance difference between the varicocele and without varicocele groups within the infertile group. This result is in accordance with the studies below that indicate the measurement of seminal fluid fructose does not contribute to the diagnosis of infertility or infection, because its discriminating power is lower than that of the ejaculate volume which is equally dependent on seminal vesicle function [36, 37]. The relationship between seminal fructose concentration and sperm characteristics was investigated in semen of 150 infertile men without evidence of disturbances in the seminal function. The result of this study is in accordance with other studies which pointed out that seminal fructose levels did not reflect the extension of prostate vesiculoepididymitis [38]. It is concluded that seminal fructose levels detected in the routine of semen analysis give no information on the clinical usefulness in defective sperm formation [36]. Therefore, in conclusion, estimation of seminal fructose fluid is not an efficient marker for the fertility.

Sperm density, viability, pH, motility (active progressive, weak progressive, sluggish, and immotile), morphology (normal shape, oval, coiled, round-head, double-head, pin-head, tapering, mid-piece defects, small, large, tail defects, and combined defects), leukocytes, viscosity, immature germ cells, and sperm autoantibodies were assessed in different levels of seminal fructose, testosterone, and progesterone concentrations, as shown in Table 1. However, none of the sperm characteristics analyzed had shown statistically significant differences among the study groups. It is concluded that seminal fructose levels detected in the routine of semen analysis give no information on the clinical usefulness in defective sperm formation.

One of the hormonal measurements in infertile males, both from the diagnostic and prognostic point of view, is the steroid hormones. In the present study, it has been observed that in most cases, there was an extraordinary increase in the levels of testosterone and progesterone, which might be the cause of fertile males. This argument is in agreement with an earlier study [39], in which it was reported that androgen production by the prostate may be markedly increased in some normal states, usually correlated with fertility. Upon the basis of the findings generated from the study, it can be safely concluded that elevated testosterone levels are indeed significantly responsible for fertility in men, as they were observed in more than one-third of the studied cases. Although some studies have claimed that serum testosterone levels demonstrate no relationship to sperm concentration or testicular biopsy [40, 41], others suggest that testosterone may assume a critical role in both the morphological development and reproductive function in males [42, 43]. Infertile patients without varicocele show very low levels of serum testosterone, as compared to patients with varicocele which appeared statistically significant. Their tissues may indicate severe seminiferous tubule atrophy, sclerosis, and Leydig cell hyperplasia. These findings imply that testosterone levels may predict whether normal spermatogenesis is occurring within the seminiferous tubules of infertile patients. However, testosterone elevation was not found to be age dependent. Since testosterone elevation always has severe indirect effects, it is suggested that in order to encounter the type of infertility this hormone causes, special efforts should be made to pinpoint the disturbance that actually is the cause of elevation in this hormone for the particular patient. 

Antisperm antibodies impair a man's fertility potential primarily by impairing sperm transport through the female reproductive tract. This immunologic factor was diagnosed when 10%–90% of motile sperms were attached to RBCs (Table 1). In this study, the prevalence of ASA among infertile males with varicocele was found to be lower than males without varicocele and corresponds to the expected prevalences present in other studies [12, 44]. These results postulated that the presence of ASA is of a little relevance in varicocele-associated infertility. However, data about the influence of varicocele on ASA formation are contradictory. Surgical correction of varicocele did not show any significant differences in semen parameters in men with or without ASA [45]. Although 50% of men with clinically palpable varicoceles were documented to have positive ASA [46], but the clinical significant of ASA is low and does not influence infertility prognosis [47]. Theoretically, in patients with varicocele, the testes suffers deleterious tissue effect which sometimes leads to a complete atrophy that attributed to the prolonged venous stasis and hyperthermia and might induce antibody formation [48]. In addition, reduced E-cadherin and *α*-catenin expression at the junctions between adjacent Sertoli cells in varicocele cases might also lead to a disruption of blood-testis barrier and the production of antibodies [49]. The debate over ASA and varicocele will continue as a number of questions remain to be answered, such as the exact role of ASA in infertility, which method of ASA testing is optimal for screening and final determination, what threshold levels of ASA are significant, which part on spermatozoa are involved in binding, how ASA interfere with the different steps in fertilization process, and what is the best method used for treatment of cases with ASAs-related infertility.

ASA showed some significant inverse relation with ages, durations of infertility, and viscosity. The importance of semen viscosity lies in the fact that the spermatozoa are tangled in the mucoid mass in the semen and prevented from migrating into the cervical tract to ascend to the site of fertilization. Seminal viscopathy was shown to be associated with male infertility [50]. We investigated the relations between semen viscosity and the presence of ASA (Table 2). A significant inverse relation between ASA and semen viscosity was revealed, and semen with ASA showed normal viscosity. This finding was not in agreement with other studies which cited to the correlation of ASA and semen hyperviscosity [51]. Local ASA stimulate interferon *γ* production, which plays, a role in enhancing directly phagocytic cells to produce hydrolyase, lipase, and esterase and indirectly by phosphor elation of proteins through activation of certain enzymes such as protein kinase. All these events participate in liquefying viscous semen and allowed it to appear with normal viscosity [52]. 

A significant correlation was observed between ASA and testosterone levels among study cases. This fact was clarified in Figure 1, in which testosterone level among infertile males without varicocele and positive for ASA was shown to be significantly higher than its level present in varicose infertile and fertile males. This is due to the risk factors for developing antisperm antibodies including a history of testicular injury, torsion, vasectomy/reversal, or infection. Alteration of steroid hormone levels such as testosterone in the seminal plasma of males with ASA has been mentioned in certain articles which suggest a relationship between testicular steroid hormone levels and autoimmunity to sperm antibodies [7]. Exogenous administration of estradiol in the rats significantly increased the IgA and IgG type of antibodies in the uterus, suggesting estradiol-dependent development of specific antibodies in the genital tract [53]. Steroids, such as estrogen and progesterone, are known to regulate lymphocyte proliferation through cytokine action [54] and participate in humoral immune responses by modulating antibody synthesis [55]. Thus, it could be said that higher testosterone levels may be responsible for higher levels of antibodies in the seminal fluids which might ultimately inhibit the motility of spermatozoa. We can concluded that high levels of testosterone stimulate the humoral immune response, and hence ASA production which causes a reduction in sperm motility.

Due to the significance of testosterone hormone in relation to males' infertility, it should invariably become a regular part of the hormonal profile recommended for men undergoing fertility assessment. Seminal fructose may not contribute to fertility through its effect on various semen parameters.

## Figures and Tables

**Table 1:** A comparison between Sperm parameters, fructose level, steroid hormones among infertile males with varicocele, without varicocele, and fertile.

Parameters	Fertile group	Infertile group		*P*-value
		Varicocele	Without varicocele	
	No = 50	No. 68	No. 82	
	Means ± SD	Means ± SD	Means ± SD	
Volume (ml)	3.54 ± 1.77	4.06 ± 1.92	3.90 ± 2.26	n.s
Count 10(6)/ml	42.10 ± 22.3	55.03 ± 77.62	36.63 ± 49.29	n.s
Viscosity	0.16 ± 0.37	0.21 ± 0.41	0.20 ± 0.46	n.s

Morphology				
Normal shape	8.12 ± 8.75	6.91 ± 6.58	7.46 ± 7.92	*
Slightly abnormal	16.48 ± 12.09	21.35 ± 11.09	17.63 ± 1284	*
Coiled	19.20 ± 17.96	13.71 ± 10.53	7.93 ± 7.65	*
Severe abnormal	56.28 ± 20.24	52.15 ± 20.79	53.36 ± 27.25	n.s

Motility				
Active progressive	8.52 ± 14.09	5.26 ± 13.28	3.05 ± 7.50	*
Weak progressive	12.76 ± 11.17	8.82 ± 12.67	7.88 ± 10.41	*
Sluggish	28.56 ± 1 17.02	35.71 ± 1 21.94	34.71 ± 1 26.54	n.s
Immotile	50.48 ± 19.36	44.56 ± 23.55	40.26 ± 28.29	n.s

Viability	50.48 ± 19.36	44.56 ± 23.55	40.26 ± 28.29	*
Fructose mg/dl	332.08 ± 172.43	359.65 ± 220.64	315.66 ± 118.49	n.s
Testosterone ng/dl	367.5 ± 21.8	380.74 ± 24.331	365.9 ± 16.55	*
Progesterone ng/ml	0.357 ± 0.0306	0.325 ± 0.243	0.341 ± 0.022	n.s

*P ≤  .05 (Significant)

n.s (Non-Significant)

**Table 2:** Relationship between autoantisperm antibodies and sperm viscosity.

Sperm viscosity	Antisperm antibodies		Total
	ASA negative	ASA positive	
Normal viscosity	114 (57%)	50 (25%)	164 (82%)
Viscous semen	30 (15%)	4 (2%)	34 (17%)
Highly viscous	2 (1%)	0 (0%)	2 (2%)
